# An Unusual Case

**Published:** 1890-01

**Authors:** Thomas K. Leonard

**Affiliations:** Blakely, Ga.


### AN UNUSUAL CASE.

Mrs. Rosannah W. Matteson, wife of Rev. Charles S. Matteson, a one-arm Yankee soldier, now living in Blakely, Georgia, gave birth to a male child in Georgetown, Quitman county, Georgia, on the 26th of November, 1888. Nothing unusual occurred in the time of an ordinary labor, but she was safely delivered, and the placenta came away in due time, but not a drop of blood escaped, either before or after the birth of the child, or after the expulsion of the placenta ; nor was there any blood, or even colored fluid, which passed away at any time, but a free discharge of colorless watery fluid.

I report this case upon this remarkable *feature*, and ask the question : Was there ever a similar case ?

All the facts in this case are obtained from Rev. Mr. Matteson and his wife, who are reliable, and who have given them to me in person. Mrs. M. has now the appearance of being nearly exsanguine, and, from her history, has always been in remarkably feeble health.

The little boy, though feeble for a considerable time, is now doing well, and growing finely.

THOMAS K. LEONARD, M. D.

*Blakely, Ga., December 3d, 1889.*

